# Keggin-Type Polyoxometalates as a Systematic Antiviral Scaffold: Activity, Cytotoxicity, and Solution Stability across Heteroion and Addenda Metal Variations

**DOI:** 10.1021/acs.jmedchem.6c01515

**Published:** 2026-08-17

**Authors:** Judith Füllborn-Ott, Aliona Dobrova, Nadiia I. Gumerova, Ingo Ott, Annette Rompel

**Affiliations:** † Institute of Medicinal and Pharmaceutical Chemistry, 26527 Technische Universität Braunschweig, Beethovenstr. 55, Braunschweig 38106, Germany; ‡ 27258 Universität Wien,Fakultät für Chemie, Institut für Biophysikalische Chemie, 1090 Wien, Austria

## Abstract

Polyoxometalates (POMs) are promising antiviral agents, yet systematic structure–activity relationships remain scarce and biological stability is rarely assessed. Here we report the antiviral activity, cytotoxicity, and solution speciation of 11 Keggin-type POMs spanning three compositional series—PV_*x*_W_12–*x*_, PV_*x*_Mo_12–*x*_, and SiMo_*x*_W_12–*x*_ (*x* = 0–3)—alongside lacunary derivatives, and simple metal salt controls. Multinuclear NMR spectroscopy (^31^P, ^51^V, ^183^W) in cell culture medium under assay conditions revealed a direct correlation between cluster integrity and antiviral potency: compounds that decompose are uniformly inactive while structurally stable or partially transformed clusters show concentration-dependent inhibition. The silicomolybdotungstate series proved most promising, with [SiW_12_O_40_]^4–^ and [SiMoW_11_O_40_]^4–^ achieving IC_50_ values of 26.0 and 24.9 μM against HCoV-OC43. Antiviral potency correlates with overall cluster charge, with 4– anions consistently outperforming their 3– and 6– analogues—an observation rationalized through the superchaotropic ion concept.

## Graphical abstract

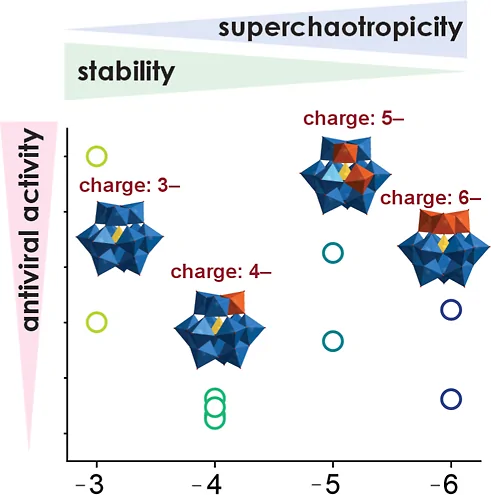

## 1 Introduction

Polyoxometalates (POMs) are discrete, soluble metal–oxygen polyanions formed by the self-assembly of early transition metals (mostly W, Mo, and V) under acidic aqueous conditions.^1^ Their biological activity has been recognized for over four decades, with early reports by Yamase and co-workers demonstrating antitumor and antiviral effects of tungstate-based clusters in the 1980s.^2,3^ The landmark review by Rhule, Hill, Judd, and Schinazi consolidated this nascent field, cataloguing POM activity against a broad panel of viruses including human immunodeficiency viruses (HIV), herpes simplex virus (HSV), and influenza, and articulating the first mechanistic hypotheses centered on electrostatic interactions between the highly charged POM anion and viral surface proteins.^4^ Since then, interest in POMs as antivirals has expanded substantially, driven by their structural tunability, synthetic accessibility, and a resistance profile that is hypothesized to differ from that of classical small-molecule antivirals owing to their multisite, noncovalent mode of interaction with viral targets.^5^

More recently, the intersection of POM chemistry and medicinal chemistry has matured into a recognized subdiscipline,^6^ with systematic efforts to define structure–activity relationships and improve selectivity indices. Bijelic, Aureliano, and Rompel provided a comprehensive survey of anticancer^7^ and antibacterial^8^ POM research spanning three decades, highlighting the recurring observation that biological potency is sensitive to cluster nuclearity, surface charge density, and heteroion identity, yet noting that rigorous structure–activity relationship studies remain scarce relative to the breadth of biological screening data. The COVID-19 pandemic renewed urgency in this area, prompting several groups to evaluate POM libraries against SARS-CoV-2 and other respiratory pathogens.^9^ While antiviral POMs have been shown to interact with viral surface proteins and interfere with early stages of infection, the molecular target identity, the mechanism by which a large polyanionic cluster accesses it in a cellular environment, and the question of which solution species is actually responsible for inhibition all remain unresolved. Addressing all of these simultaneously lies beyond the scope of any single study, however, the present work makes a defined contribution by establishing that intact cluster architecture is a prerequisite for antiviral activity. This is not merely a question of experimental rigor, it reflects a fundamental physicochemical challenge inherent to POM chemistry. Unlike classical organic drugs, POMs are metastable species whose integrity depends critically on pH, ionic strength, concentration, and the presence of competing ligands, all of which differ dramatically between a dilute aqueous stock solution and cell culture medium.^10–12^ Crans and co-workers, established that POM speciation in solution is dynamic and that decomposition products (free oxoanions, lacunary species, and small polynuclear fragments) can differ substantially in charge, geometry, and biological activity from the parent cluster.^13,14^ Solution-state multinuclear NMR spectroscopy, principally ^31^P, ^51^V, and ^183^W, has emerged as the most powerful tool for speciation analysis under biologically relevant conditions, enabling direct identification and quantification of intact clusters, decomposition intermediates, and degradation products in complex media.^10^ Our group has previously developed and validated multinuclear NMR protocols for the systematic mapping of POM speciation in cell culture media,^11,12,15^ and the present study builds directly on this methodological foundation to interpret antiviral activity in terms of the species actually present during biological assays.

Among the diverse POM structural families, the Keggin anion occupies a privileged position in medicinal chemistry investigations by virtue of its exceptional synthetic accessibility, well-defined geometry, and tolerance for systematic compositional variation.^1^ The primary structure [XM_12_O_40_]^n–^ consists of a central heteroion tetrahedron (X = P^V^, Si^IV^, Ge^IV^, B^III^, among others) encapsulated within a cage of 12 edge- and corner-sharing {MO_6_} octahedra (M = W, Mo), yielding a highly symmetric cluster with *T*_*d*_ symmetry in its α-isomeric form. This architecture offers three independent chemical handles for rational modification. The heteroion X determines the overall cluster charge and influences the electrostatic potential at the molecular surface; the addenda metal M governs redox behavior, Lewis acidity, and solubility; and partial substitution of M by vanadium or other transition metals introduces additional redox activity and lowers overall symmetry, generating families of mixed-metal derivatives. These handles can be varied independently and in combination, making the Keggin framework an unusually tractable inorganic scaffold for structure–activity relationship studies. The present study exploits this systematically by examining three isostructural series: PV_*x*_W_12–*x*_ (*x* = 0–3), PV_*x*_Mo_12–*x*_ (*x* = 0–3), and SiMo_*x*_W_12–*x*_ (*x* = 0, 1, 3) (Figure 1), alongside lacunary derivatives and simple metal salt controls, thereby spanning the key compositional axes in a controlled manner. Additionally, Keggin-type POMs have been demonstrated to behave as superchaotropic anions, since they are large, polarizable, weakly hydrated species that interact favorably with hydrophobic interfaces and can partition into lipid bilayers.^16–18^

**Figure 1:**
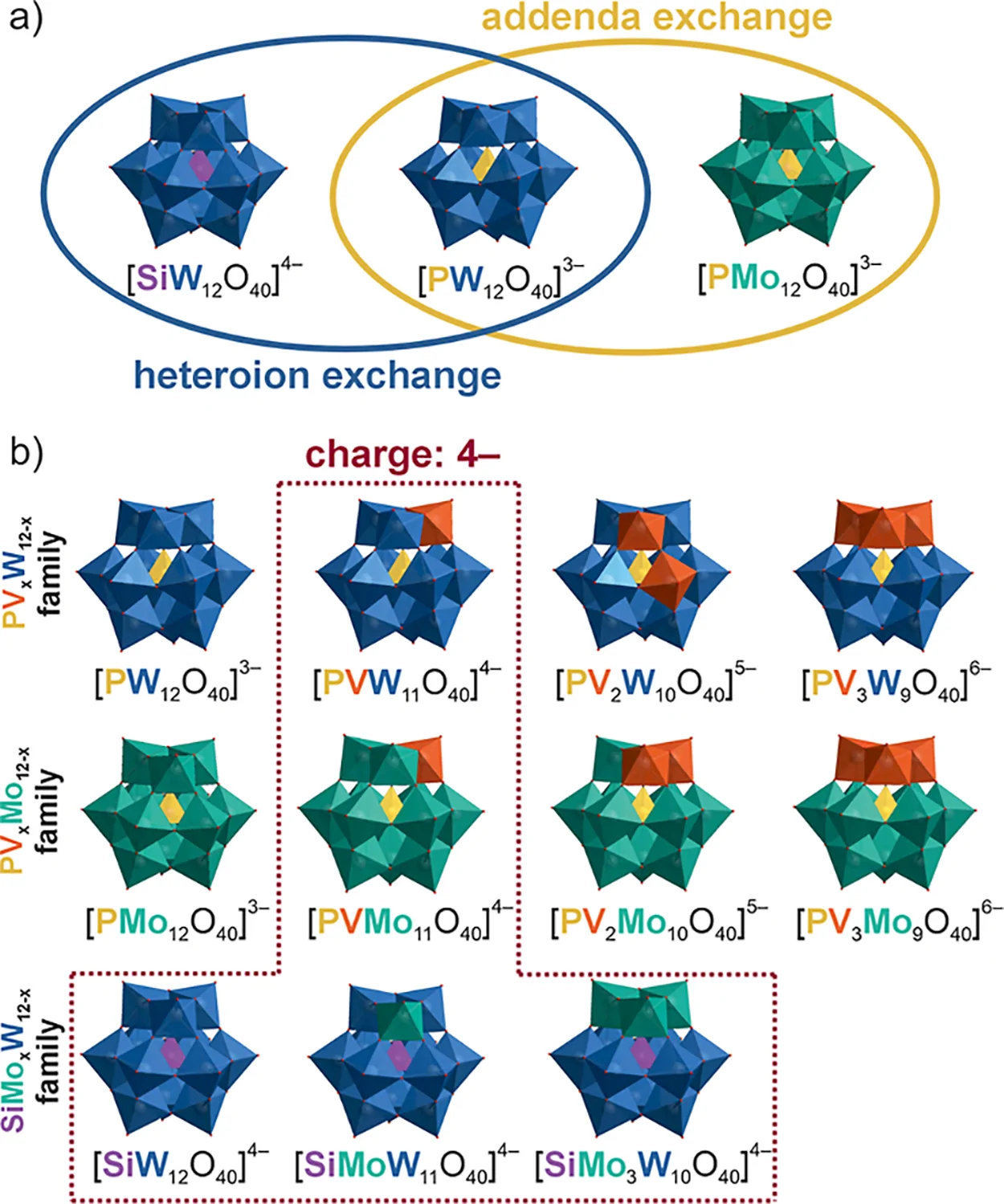
Design strategy and compound library of Keggin-type polyoxometalates investigated in this study. (a) The two principal structural axes: heteroion exchange (Si vs P, blue ellipse) and addenda metal exchange (W vs Mo, gold ellipse), illustrated by the parent clusters [SiW_12_O_40_]^4–^, [PW_12_O_40_]^3–^, and [PMo_12_O_40_]^3–^. (b) The full library of 11 Keggin anions spanning three isostructural series: PV_*x*_W_12–*x*_ (*x* = 0–3, top row), PV_*x*_Mo_12–*x*_ (*x* = 0–3, middle row), and SiMo_*x*_W_12–*x*_ (*x* = 0, 1, 3, bottom row). The dashed red box highlights compounds sharing an overall cluster charge of 4–. Color code: W-centered octahedra (blue), Mo-centered octahedra (teal), V-substituted positions (orange), heteroions—P (gold), Si (magenta).

The overarching objective of this work is to establish rigorous structure–activity–stability relationships for Keggin-type POMs as antiviral agents, using HCoV-OC43 as a well-established coronavirus model system, by integrating two complementary experimental approaches. First, we report the systematic antiviral screening and cytotoxicity profiling of a rationally assembled library comprising 11 Keggin-type clusters spanning three isostructural series [PV_*x*_W_12–*x*_O_40_]^(3+x)–^ (*x* = 0–3), [PV_*x*_Mo_12–*x*_O_40_]^(3+x)–^ (*x* = 0–3), and [SiMo_*x*_W_12–*x*_O_40_]^4–^ (*x* = 0, 1, 3), together with lacunary derivatives [PM_11_O_39_]^7–^ (M = Mo or W), a decavanadate [V_10_O_28_]^6–^ control, and simple metal salt controls to exclude heteroion-mediated effects. Second, and critically, the solution stability of each compound is assessed by multinuclear NMR spectroscopy (^31^P, ^51^V, and ^183^W) in cell culture medium under conditions directly matching those of the antiviral assay, allowing observed IC_50_ values to be interpreted in terms of the actual species present during the biological experiment.

## 2 Results and Discussion

### 2.1 Antiviral Activity and Cytotoxicity

All compounds were evaluated for antiviral activity against human coronavirus HCoV-OC43, a widely used surrogate for the highly pathogenic SARS-CoV-2 that allows biosafety level 2 handling while recapitulating key features of coronavirus replication. IC_50_ and CC_50_ values are summarized in Table 1 and Figures S16–S21. Three reference compounds were included to contextualize the activity of the Keggin library. Remdesivir, an FDA-approved nucleotide analogue and RdRp inhibitor, was chosen as a clinically validated antiviral benchmark with a well-defined intracellular mechanism of action, allowing direct comparison with our compounds in the same assay. K_7_[PTi_2_W_10_O_40_] was selected as a structurally related POM reference with reported broad-spectrum antiviral activity against HCoV-OC43 and other respiratory viruses.^19^ K_11_H­[(VO)_3_(SbW_9_O_33_)_2_] was included as a non-Keggin POM of known potent antiviral activity,^20^ serving as an upper benchmark for what POM-based inhibition can achieve. All IC_50_ and CC_50_ values for reference compounds were determined in the present study under identical assay conditions. Under the assay conditions employed, remdesivir and K_11_H­[(VO)_3_(SbW_9_O_33_)_2_] showed strong antiviral activity (IC_50_ = 0.07 and 2.8 μM, respectively). K_7_[PTi_2_W_10_O_40_] displayed only weak inhibition under the present assay conditions. Direct comparison with literature^19^ values is not meaningful, as the assay principle, host cells, exposure periods and other relevant experimental parameters differed substantially. Critically, the speciation data reported here do not merely contextualize the biological results, they indicate which IC_50_ values reflect the activity of a chemically defined intact species and which reflect the biological outcome of compound decomposition. This distinction is essential for any legitimate SAR analysis and that is absent from the majority of published POM antiviral studies.

**Table 1:** Antiviral Activity (IC_50_) and Cytotoxicity (CC_50_) of Keggin-type Polyoxometalates, Extended POM Derivatives, Metal Salt Controls, and Reference Compounds against HCoV-OC43^a^

compound	abbreviations used in this study	compound charge in solid state	IC_50_ [μM]	CC_50_ [μM]	SI
Na_3_[PW_12_O_40_]	PW_12_	–3	200.5 ± 14.2	>500	>2
H_3_[PMo_12_O_40_]	PMo_12_	–3	>500	>500	
K_4_[PVW_11_O_40_]	PVW_11_	–4	32.3 ± 2.2	>200	>6
H_4_[PVMo_11_O_40_]	PVMo_11_	–4	63.0 ± 7.3	>200	>3
K_4_[SiW_12_O_40_]	SiW_12_	–4	26.0 ± 2.6	>250	>9
K_4_[SiMoW_11_O_40_]	SiMoW_11_	–4	24.9 ± 2.0	>250	>10
K_4_[SiMo_3_W_9_O_40_]	SiMo_3_W_9_	–4	47.1 ± 1.8	>500	>10
H_5_[PV_2_Mo_10_O_40_]	PV_2_Mo_10_	–5	166.4 ± 34.1	>500	>3
Cs_5_[PV_2_W_10_O_40_]	PV_2_W_10_	–5	325.7 ± 82.8	>500	>1
Cs_6_[PV_3_W_9_O_40_]	PV_3_W_9_	–6	61.6 ± 14.1	>500	>8
H_6_[PV_3_Mo_9_O_40_]	PV_3_Mo_9_	–6	222.9 ± 16.1	>500	>2
Na_7_[PW_11_O_39_]	PW_11_	–7	299.7 ± 50.9	>500	>1
Na_7_[PMo_11_O_39_]	PMo_11_	–7	428.1 ± 55.9	>500	>1
Na_2_K_4_[V_10_O_28_]	V_10_	–6	151.9 ± 41.9	>500	>3
Na_2_MoO_4_	–	–	>500	>500	
Na_2_SiO_3_	–	–	>500	>500	
Na_2_WO_4_	–	–	>500	>500	
Na_3_VO_4_	–	–	>500	>500	
K_7_[PTi_2_W_10_O_40_]	–	–7	109.4 ± 32.4	>200	>1
K_11_H[(VO)_3_(SbW_9_O_33_)_2_]	–	–12	2.8 ± 1.3	>100	>35
Remdesivir	–	–	0.07 ± 0.03	>10	>142

a Values are given as mean ± SD (*n* = 3–4). As CC_50_-values could not be determined, the selectivity indices (Supporting Information) were calculated with the maximum tested concentration and represent the lower boundary. The nominal cluster charge refers to the intact anion in the solid state; solution speciation under assay conditions is discussed in the text. Compounds are grouped by the anion charge.

The tested POMs displayed IC_50_ values spanning more than 1 order of magnitude, from 24.9 μM (SiMoW_11_) to above 500 μM (PMo_12_). A clear dependence of antiviral potency on the overall cluster charge was observed: all compounds carrying a 4– charge showed IC_50_ values below 65 μM, while compounds with charges of 3–, 5–, 6–, and 7– were substantially less active (IC_50_ > 150 μM), with the sole exception of PV_3_W_9_ (IC_50_ = 61.6 μM). The most potent Keggin-type POMs were SiW_12_ and SiMoW_11_, with IC_50_ values of 26.0 and 24.9 μM (approximately 350-fold less potent than remdesivir IC_50_ = 0.07 μM), respectively. Importantly, none of the tested compounds showed cytotoxicity against host cells within the studied concentration range (Table 1), confirming that the observed inhibition represents genuine antiviral activity rather than a cytotoxic artifact with SIs higher than 6, 9, and 10 for the most active species.

To probe whether the observed activity could be attributed to fragment species or free metal ions rather than the intact cluster, a series of related lacunary POMs and simple metal salt precursors were included. The lacunary compounds PW_11_ (IC_50_ = 299.7 μM) and PMo_11_ (IC_50_ = 428.1 μM) showed comparable activity to the solutions of their parent Keggins PW_12_ (IC_50_ = 200.5 μM) and PMo_12_ (IC_50_ > 500 μM), respectively. The decavanadate V_10_ showed moderate activity (IC_50_ = 151.9 μM), while all simple metal salts, Na_2_MoO_4_, Na_2_SiO_3_, Na_2_WO_4_, and Na_3_VO_4_, were entirely inactive (IC_50_ > 500 μM), ruling out free metal ion release as the source of antiviral potency and establishing intact cluster architecture as a prerequisite for biological activity.

In antiviral drug research time-of-addition assays are designed to provide information when in the viral life cycle a drug is active. By adding the test compound at different time points relative to the infection (i.e., before, simultaneously, or after), likely mechanisms of action can be indirectly inferred. The most active species of each POM family (PVW_11_, PVMo_11_ and SiMoW_11_), the inactive POM PMo_12_, the reference compounds K_11_H­[(VO)_3_(SbW_9_O_33_)_2_] and remdesivir were chosen for these additional antiviral studies (Table 2, Figure 2).

**Table 2:** Antiviral Activity (IC_50_) and Time-of Addition Results of Selected most Active Species (PVW_11_, PVMo_11_, and SiMoW_11_), Inactive Control PMo_12_ and References K_11_H­[(VO)_3_(SbW_9_O_33_)_2_] and RdRp-Inhibitor Remdesivir (*n* = 3–4)

compound	abbreviations used in this study	IC_50_ [μM] Pre	IC_50_ [μM] Co	IC_50_ [μM] post
K_4_[PVW_11_O_40_]	PVW_11_	>200	61.3 ± 8.2	86.8 ± 11.6
H_3_[PMo_12_O_40_]	PMo_12_	>500	>500	>500
H_4_[PVMo_11_O_40_]	PVMo_11_	>200	59.3 ± 6.5	163.6 ± 30.5
K_4_[SiMoW_11_O_40_]	SiMoW_11_	>250	54.6 ± 7.5	72.6 ± 13.8
K_11_H[(VO)_3_(SbW_9_O_33_)_2_]	–	>100	12.7 ± 4.3	8.7 ± 2.8
remdesivir	–	0.6 ± 0.04	0.4 ± 0.2	0.05 ± 0.01

**Figure 2:**
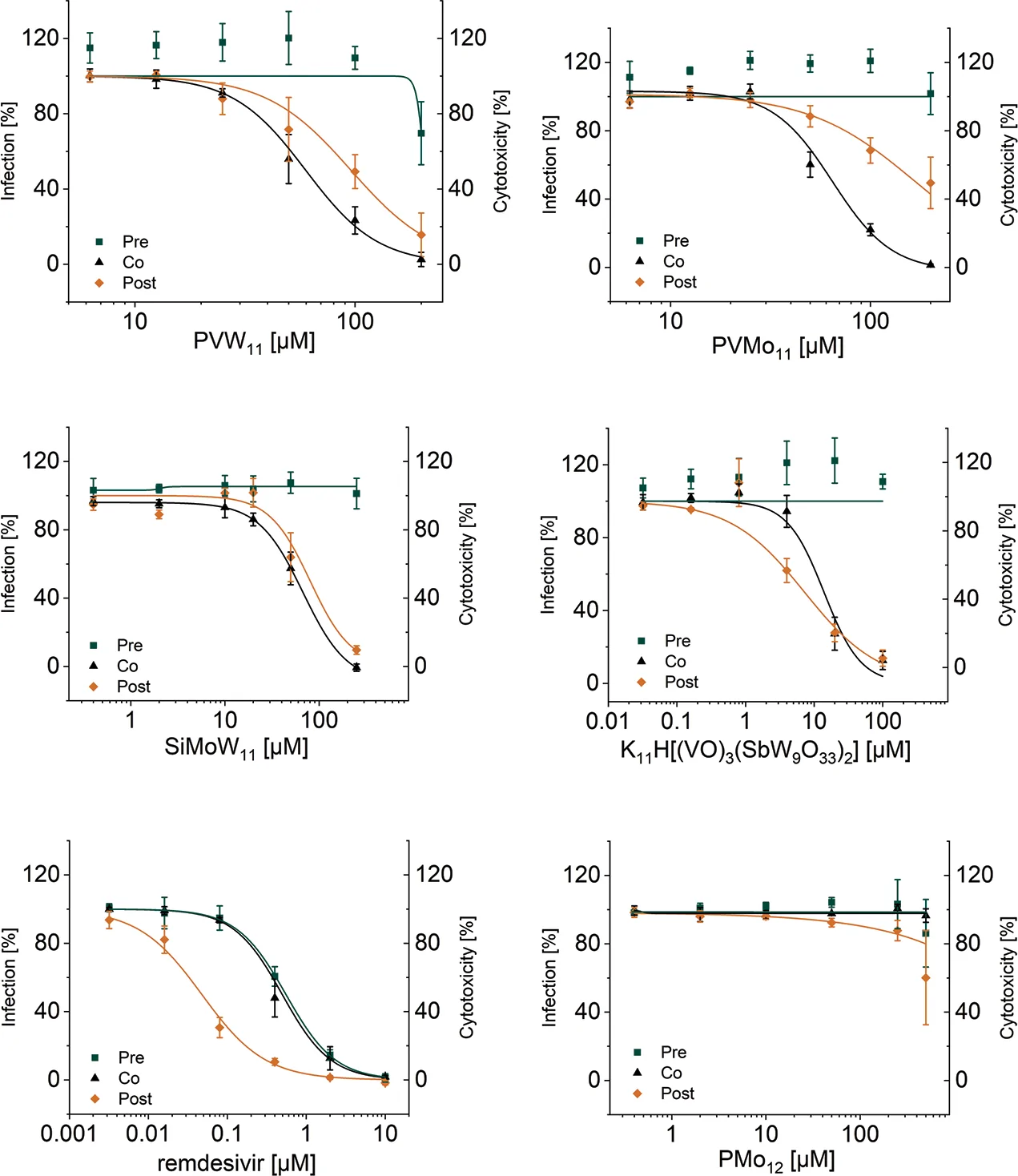
Results of time-of-addition experiments for the most active species of each group (PVW_11_, PVMo_11_ and SiMoW_11_), inactive species PMo_12_ and references K_11_H­[(VO)_3_(SbW_9_O_33_)_2_] and RNA-dependent RNA polymerase-inhibitor remdesivir (*n* = 3–4). Pretreatment (Pre) curves are shown in dark green, cotreatment (Co) in black and posttreatment (Post) in orange. For IC_50_ and CC_50_ values (n=3–4) see Table 2.

As expected for an RNA-dependent RNA polymerase (RdRp) inhibitor acting on intracellular replication, reference remdesivir showed 10-times stronger activity in post-treatment setup compared to pre- and cotreatment with an IC_50_ of 0.05 μM. The inactive control PMo_12_ showed no activity in the time-of addition assay as expected.

All three active POMs were inactive up to the highest tested concentration under pretreatment conditions. In cotreatment setup, PVW_11_, PVMo_11_, and SiMoW_11_ exhibited lower IC_50_ values compared to post-treatment conditions. This effect was most pronounced for PVMo_11_, which reached comparable IC_50_ values under cotreatment conditions (IC_50_ (Co) = 59.3 μM) and the original antiviral assay that is based on a continuous treatment (IC_50_ = 63.0 μM, see Table 1). All other POMs had lower activity in the time-of-addition assay than in the previous continuous assay (see Table 1). The inactivity of PVW_11_, PVMo_11_, and SiMoW_11_ in the pretreatment setup makes a mechanism of action as entry inhibitor by blocking cellular receptors unlikely. On the other hand, the consistent pattern of the three POMs to be most active in the time-of-addition assay when added simultaneously with infection and to show lower activity in the post-treatment setup, strongly suggests that they trigger virucidal effects or reduce entry by blocking viral entry receptors. Lower, but continuous activity in the post-treatment setup may indicate the mechanism of action extends beyondthe entry stage. The reference compound K_11_H­[(VO)_3_(SbW_9_O_33_)_2_] showed a different profile, retaining substantial activity under both co- and post-treatment conditions (IC_50_ = 12.7 and 8.7 μM, respectively), suggesting a partially overlapping but distinct mechanism relative to the Keggin series. The noninterference of the selected POMs with the viral entry stage on the cellular side is unexpected, given that this mechanism has been proposed for other POMs, including K_7_[PTi_2_W_10_O_40_];^19,21,22^ however, activity of POMs against viral host cell receptors^9^ and enzymatic targets relevant for postentry viral replication have been reported for several examples,^9,23^ suggesting an overall multimodal mechanism for antiviral POMs.

### 2.2 Solution Stability and Speciation in Biological Media

To interpret the antiviral data in mechanistically meaningful terms, the solution behavior of all 11 Keggin anions was assessed by multinuclear NMR spectroscopy (^31^P, ^51^V, ^183^W) under three conditions: pure H_2_O, freshly prepared DMEM (Dulbecco’s Modified Eagle Medium) supplemented with 2% FCS (Fetal Calf Serum) and 50 mg/L gentamicin (fresh DMEM), and the same medium composition after 24 h incubation at 37 °C (incubated DMEM). The latter condition directly mirrors the duration and temperature of the antiviral assay, ensuring that speciation data are directly comparable to the biological results. pH measurements carried out in parallel revealed pronounced family dependent behavior (Figure S1): 1 mM solutions of the PV_*x*_W_12–x_ series (Figure 1b) exhibited near-neutral pH in H_2_O, with slightly lower values for the less negatively charged members and were effectively buffered to pH ∼ 7 in both fresh and incubated DMEM. By contrast, PV_*x*_Mo_12–*x*_ (Figure 1b) solutions were strongly acidic in H_2_O (pH ∼ 2), a consequence of the lower solution stability of the molybdate Keggin under unbuffered conditions and were only partially corrected by DMEM to pH 5–6. The SiMo_*x*_W_12–*x*_ family, examined separately, reached pH ∼ 7 in DMEM across all members. These pH profiles already foreshadow the stark stability differences observed by NMR. The signals were assigned based on the literature.^11,12,24–27^

#### 2.2.1 PV_*x*_W_12–*x*_ Series

Within the tungstate series (Figure 1b), stability increased with vanadium content. The parent phosphotungstate [PW_12_O_40_]^3–^ (PW_12_) was already largely decomposed in H_2_O (∼30% intact by ^31^P NMR, Figure S2), converting entirely to lacunary [PW_11_O_39_]^7–^ (−11.8 ppm) and free phosphate (2.0 ppm) in DMEM. The instability of PW_12_ already at the stock solution stage provides a straightforward explanation for its poor antiviral activity (IC_50_ = 200.5 μM): the compound is largely decomposed before it reaches the biological target. The monosubstituted [PVW_11_O_40_]^4–^ (PVW_11_) was fully intact in H_2_O by ^31^P NMR, appearing as a single sharp resonance at δ –14.9 ppm characteristic of the intact Keggin (Figure S3), while ^51^V NMR showed a broadened signal at δ –556 ppm consistent with intact PVW_11_ upon normalization (Figure S4). In DMEM, the ^31^P signal was no longer detectable, most likely due to paramagnetic relaxation enhancement of the centrally positioned phosphorus caused by partial V^5+^ → V^4+^ reduction within the cluster framework. ^51^V NMR, which retains sensitivity to the remaining diamagnetic V^5+^ centers, confirmed the presence of intact PVW_11_ upon normalization, indicating that the cluster is partially preserved under assay conditions (Figure S4). The comparatively good antiviral activity of PVW_11_ (IC_50_ = 32.3 μM, the lowest in this series) is consistent with the presence of at least a fraction of intact Keggin anion during the assay. The disubstituted [PV_2_W_10_O_40_]^5–^ (PV_2_W_10_) existed as a mixture of α-, β-, and γ-isomers in H_2_O (Σ = 74.5% by ^31^P NMR, Figure S5), with decomposition to decavanadate [V_10_O_28_]^6–^ and Lindqvist-anion [VW_5_O_19_]^5–^ identified by ^51^V NMR accelerating upon incubation in DMEM (Figures S5 and S6), in line with its negligible activity (IC_50_ = 325.7 μM). The trisubstituted [PV_3_W_9_O_40_]^6–^ (PV_3_W_9_) was the most stable (Figures S7 and S8) member of this series with a single sharp ^31^P signal at —13.5 ppm throughout all conditions and with only minor leaching of small oligomeric vanadate species—divanadate [V_2_O_7_]^4–^, trivanadate [V_3_O_9_]^3–^, and tetravanadate [V_4_O_12_]^4–^, collectively referred to as V_2_–V_4_—detected by ^51^V NMR in DMEM. Its moderate activity (IC_50_ = 61.6 μM) despite excellent stability suggests that structural integrity alone is insufficient for potency and that surface charge distribution plays an additional role.

#### 2.2.2 PV_*x*_Mo_12‑*x*_ Series

The phosphomolybdate family (Figure 1b) was uniformly less stable, consistent with the greater lability of the Mo–O framework.^1^ The phosphomolybdate [PMo_12_O_40_]^3–^ (PMo_12_) decomposed completely in DMEM as evidenced by ^31^P NMR spectroscopy (Figure S9) to free phosphate and [P_2_Mo_5_O_23_]^6–^ (2.7 ppm), correlating with its negligible activity (IC_50_ = > 500 μM). Among the vanadium-substituted molybdates, [PVMo_11_O_40_]^4–^ (PVMo_11_) proved the most resilient (Figures S10 and S11) and, notably, the most active compound in this family (IC_50_ = 63.0 μM). ^51^V NMR revealed that 73–75% (signal at δ ∼ −530 ppm, quantified by integration relative to total vanadium signal (Figure S11)) of vanadium was retained in a Keggin-type V–Mo mixed species in both fresh and incubated DMEM, with no progressive decomposition. Higher vanadium substitution led to substantially more complex speciation. Both disubstituted [PV_2_Mo_10_O_40_]^5–^ (PV_2_Mo_10_) and trisubstituted [PV_3_Mo_9_O_40_]^6–^ (PV_3_Mo_9_) anions yielded complex, time-evolving mixtures of lower-vanadium Keggin species, V–Mo mixed anions, and small vanadates (V_2_–V_4_)^11^ in DMEM, with near-complete loss of intact parent cluster after incubation (Figures S12–S15). Their poor antiviral activity (IC_50_ = 166.4 and 222.9 μM, respectively) reflects this instability directly.

#### 2.2.3 SiMo_*x*_W_12‑*x*_ Series

The silicomolybdotungstate family (Figure 1b) was examined by ^183^W NMR, a nucleus particularly well suited to distinguishing Keggin isomers. The ^183^W NMR acquisition time of approximately 60 h substantially exceeds the 24 h duration of the antiviral assay, and the speciation data therefore reflect the equilibrium state of freshly prepared DMEM solutions rather than a time-matched snapshot. Time-dependent changes occurring between 24 and 60 h cannot be excluded. Additionally, POM concentration was 20 mM, which is higher than the IC_50_ range of the active compounds and was necessitated by the inherently low sensitivity of the ^183^W nucleus. ^29^Si NMR proved uninformative under the conditions employed, likely due to the unfavorable combination of low natural abundance (4.7%), poor sensitivity, and extensive signal broadening in the complex DMEM matrix. All measurements were therefore performed on freshly prepared DMEM solutions. The parent SiW_12_ displayed complete stability with an identical single ^183^W resonance in H_2_O and DMEM (δ ∼ –100 ppm, pH 6.6, Figure 3a), consistent with its robust antiviral activity (IC_50_ = 26.0 μM). The monosubstituted SiMoW_11_ displayed a characteristic six-signal ^183^W pattern in H_2_O that collapsed to a single resonance in DMEM (pH 6.9, Figure 3b), accompanied by a visible color change indicative of Mo reduction; despite this partial transformation, strong activity was retained (IC_50_ = 24.9 μM), suggesting that the SiW_12_ species formed in situ may contribute to the observed effect. The trisubstituted SiMo_3_W_9_ showed no detectable ^183^W signals in DMEM and an immediate color change upon dissolution, indicating extensive decomposition. Nevertheless, measurable antiviral activity was retained (IC_50_ = 47.1 μM), possibly through biologically active decomposition products.

**Figure 3:**
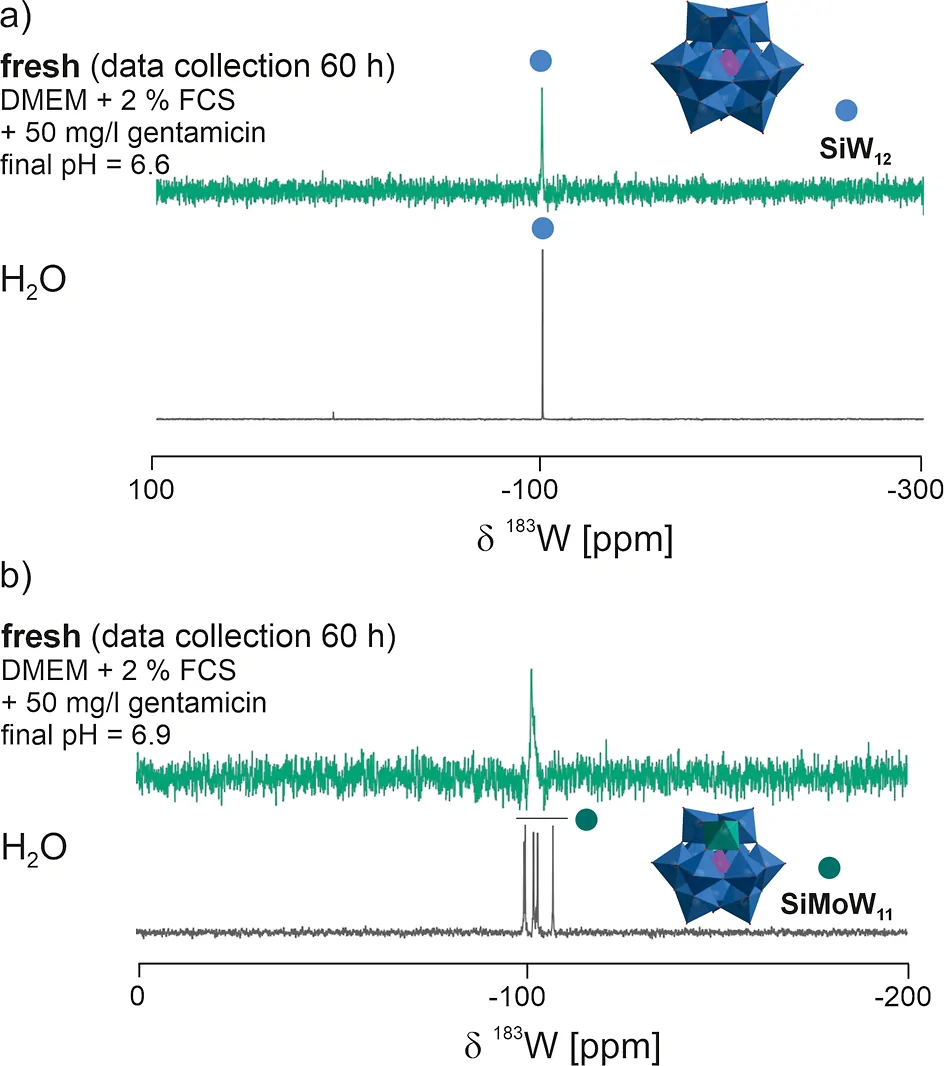
^183^W NMR spectra of (a) SiW_12_ and (b) SiMoW_11_ recorded in H_2_O (gray, bottom) and in freshly prepared DMEM +2% FCS (Fetal Calf Serum) + 50 mg/L gentamicin (green, top; data collection 60 h per sample). Color code: W-centered octahedra (blue), Mo-centered octahedra (teal), Si (magenta). Signals corresponding to individual POM species are indicated by filled circles.

#### 2.2.4 Toward a Charge-Activity Relationship: The Superchaotropic Ion as a Working Hypothesis

A striking pattern emerges when antiviral potency is linked to the overall cluster charge of the intact Keggin anion (Figure 4). The four most active compounds in the library—SiW_12_ (IC_50_ = 26.0 μM), SiMoW_11_ (IC_50_ = 24.9 μM), PVW_11_ (IC_50_ = 32.3 μM), and PVMo_11_ (IC_50_ = 63.0 μM), share an overall charge of 4–, highlighted in Figure 1b. By contrast, the 3– members PW_12_ and PMo_12_ are inactive, but this is confounded by their instability in biological media. More informative are the higher-charge (5– and 6−) members: PV_2_W_10_, PV_3_W_9_, and their molybdate analogues, which are structurally intact or partially stable in DMEM yet consistently less active than their 4– counterparts. This charge-dependent activity cliff, separating stable but weakly active 5– and 6– anions from potent 4– anions, suggests that stability alone does not govern antiviral potency and that the charge magnitude plays an independent mechanistic role. In this framework, the 4– Keggins occupy a charge Goldilocks zone: sufficiently hydrophobic in their ion–water interactions to associate with and permeate biological membranes, yet not so highly charged that electrostatic repulsion from the negatively charged membrane surface impedes partitioning. Anions carrying 5– or 6– charges would face a progressively larger desolvation penalty and stronger electrostatic barrier, reducing their capacity to act as transmembrane carriers or to access membrane-proximal viral entry machinery. This hypothesis is consistent with the observation that PV_3_W_9_, despite being completely intact in DMEM, achieves only moderate antiviral activity: its 6– charge may simply be too high to permit effective membrane interaction regardless of structural integrity. Conversely, the 3– anions PW_12_ and PMo_12_, although superchaotropic, decomposed in biological media (Figures S2 and S9). It should be noted that charge is not the only variable differing across the compound series: composition, solution stability, and counterion identity, and their individual contributions to the observed activity differences cannot be fully deconvoluted from the present data set. Systematic resynthesis of selected compounds as identical counterion salts and direct biological comparison would be required to isolate the charge effect rigorously. With these caveats in mind, the 4– charge optimum and the superchaotropic rationalization are presented here as a working hypothesis rather than an established design rule.

**Figure 4:**
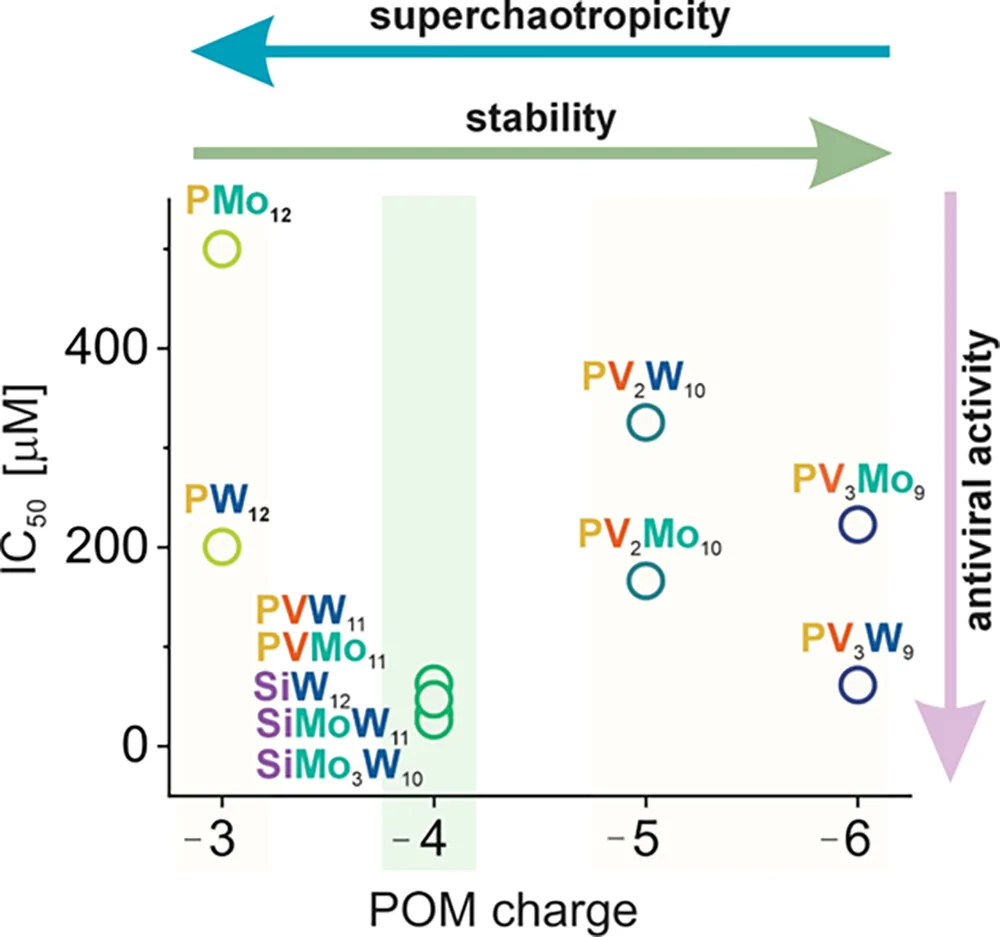
Relationship between overall Keggin anion charge and antiviral IC_50_ against HCoV-OC43. Each symbol represents one compound. The green shaded region highlights the 4– charge state, which corresponds to the lowest IC_50_ values across all three series. The opposing arrows illustrate the two competing physicochemical trends governing antiviral activity: superchaotropicity, which decreases with increasing negative charge and favors membrane partitioning, and solution stability, which increases with increasing negative charge.

The relationship between cluster integrity and antiviral activity falls into three categories. First, clusters that decompose completely in DMEM (PW_12_, PMo_12_, PV_2_Mo_10_, and PV_3_Mo_9_) are uniformly inactive, a correlation confirmed by the inactivity of all simple metal salt controls. Second, clusters confirmed intact or substantially intact by NMR—SiW_12_ (completely stable, ^183^W NMR), PVMo_11_ (73–75% intact, ^51^V NMR), and PVW_11_ (partially intact, ^51^V NMR)—show clear antiviral activity attributable to the intact Keggin anion. Third, SiMoW_11_ and SiMo_3_W_9_ represent ambiguous cases: the ^183^W pattern of SiMoW_11_ collapses to a resonance coinciding with SiW_12_ in DMEM (Figure 3), suggesting in situ retro-substitution, while SiMo_3_W_9_ yields no detectable ^183^W signal yet retains measurable activity (IC_50_ = 47.1 μM). In both cases the identity of the active species cannot be unambiguously assigned from the present data, and the conclusion that the intact Keggin cluster is universally the essential pharmacophore must therefore be qualified accordingly.

While the Keggin POMs identified here do not yet compete with optimized small-molecule antivirals in absolute potency, three independent arguments support their continued development. Their mechanism of action is orthogonal to all approved coronavirus antivirals, making them intrinsically suited to combination regimens in which synergistic effects can lower the required concentration of each component below its single-agent clinical threshold. Their multisite, noncovalent interaction mode is structurally resistant to the single-point mutations that rapidly erode conventional antiviral efficacy. Most concretely, K_11_H­[(VO)_3_(SbW_9_O_33_)_2_] demonstrates under identical assay conditions that IC_50_ = 2.84 μM is achievable within the broader POM chemotype, establishing a realistic near-term potency milestone of approximately 40-fold below remdesivir and representing a concrete target for the optimization strategies outlined below. Building on the design principles established here, several structural optimization strategies are immediately suggested by the data. First, the charge-activity relationship implies that maintaining the 4– charge window of Keggin type anions while modifying surface electrostatics and redox properties is the primary design criterion. Within the 4– charge window, the most immediately tractable inorganic optimization axis is systematic variation of the heteroion identity, for example, replacing Si^4+^ or P^5+^ with Ge^4+^ or B^3+^, while preserving the overall charge. Second, the lacunary derivatives of the active 4– Keggins, in particular [SiW_11_O_39_]^8–^ and [PW_11_O_39_]^7–^ and its monoprotonated forms, offer reactive vacant sites for grafting of metal-based antiviral pharmacophores.

To contextualize the antiviral potency of the Keggin library, K_11_H­[(VO)_3_(SbW_9_O_33_)_2_], a Krebs-type vanadotungstate sandwich complex of known antiviral activity,^3,20^ was included as a structural reference compound. With an IC_50_ of 2.8 μM, which is approximately one order of magnitude below the most active Keggin members, K_11_H­[(VO)_3_(SbW_9_O_33_)_2_] confirms that higher structural complexity and nuclearity can translate to substantially greater potency within the POM chemotype. The Keggin library, by design, was constructed to extract transferable structure–activity and structure–stability relationships rather than to optimize absolute potency, and in this respect it fully achieves its objective: it identifies cluster charge 4– as a strong candidate for the optimal charge state for antiviral activity, provides evidence that solution stability is a prerequisite for biological efficacy, and identifies the SiMo_*x*_W_12–*x*_ family as the most promising Keggin-based leads for further investigation. The outstanding potency of K_11_H­[(VO)_3_(SbW_9_O_33_)_2_] relative to all Keggin members may reflect its distinct topology and higher nuclearity, the presence of vanadyl VO^2+^ units in a coordination environment less susceptible to reduction than framework-substituted vanadium, or potentially the contribution of antimony—Sb^3+^ has independently documented antiviral properties.^28^

To place these findings in a broader therapeutic context, the IC_50_ values of the most active Keggin compounds—SiW_12_ and SiMoW_11_ at 26.0 and 24.9 μM respectively—can be compared with those of clinically used antivirals against HCoV-OC43. Remdesivir, included as an internal reference in all assays, achieved an IC_50_ of 0.07 μM under identical conditions, representing an approximately 350-fold potency advantage over the best Keggin compounds. While the Keggin POMs do not yet compete with optimized small-molecule antivirals in absolute potency, it should be noted that these are unoptimized inorganic scaffolds evaluated without any structural elaboration, that their selectivity indices are acceptable for a first-generation series, and that their mode of action is mechanistically orthogonal to existing drugs, making them candidates for combination regimens where resistance to conventional antivirals is a concern.

The most relevant pharmacokinetic consideration at this stage is solution stability under physiological conditions—assessed here by NMR speciation—which serves as the appropriate surrogate for metabolic stability. The cytotoxicity data (CC_50_ > 200–500 μM) provide the primary toxicity assessment. Renal clearance is the expected primary excretion route for highly hydrophilic, charged cluster anions, and oral bioavailability is expected to be limited, directing attention toward parenteral or inhalation administration routes consistent with historical precedent for antiviral POMs. Formal in vivo ADMET (Absorption, Distribution, Metabolism, Excretion, Toxicity) profiling of the lead compounds identified here represents an essential objective for subsequent studies.

## 3 Conclusions

This study demonstrates that the Keggin POM framework constitutes a tractable and systematically explorable inorganic scaffold for antiviral drug discovery, provided that two conditions are met: the intact cluster must be preserved under physiological conditions, and the overall anion charge must fall within a defined optimal range. By combining antiviral screening against HCoV-OC43 with multinuclear NMR speciation analysis performed under conditions directly matching those of the biological assay, we show that stability and charge are not independent but deeply interrelated determinants of activity. The silicomolybdotungstate series, and in particular SiW_12_ and SiMoW_11_, emerges as the most promising Keggin-based lead series, combining confirmed solution integrity with IC_50_ values below 30 μM, acceptable selectivity indices, and a mechanism of action at an early postentry stage of the viral life cycle that is mechanistically orthogonal to approved nucleotide analogues such as remdesivir. The charge-activity relationship rationalized through the superchaotropic ion concept provides a predictive framework for future scaffold optimization. The inactivity of all simple metal salt controls confirms that free metal ions do not account for antiviral potency; while intact cluster architecture is clearly required for activity in the majority of cases, the ambiguous speciation of SiMoW_11_ and SiMo_3_W_9_ in DMEM indicates that transformed POM-derived species may contribute to activity in certain cases, a distinction that future mechanistic studies should address explicitly. Taken together, these findings offer a methodological template, integrating rational library design, speciation-aware biological evaluation, and mechanistic time-of-addition profiling, that we propose as a standard framework for rigorous POM antiviral studies going forward.

## 4 Experimental Section

H_3_[PMo_12_O_40_] (≥99%, ACS reagent), Na_2_WO_4_·2H_2_O (≥99%, ACS reagent), Na_2_MoO_4_·2H_2_O (≥99%, ACS reagent), NaVO_3_ (≥99%, ACS reagent), Na_2_SiO_3_ (reagent grade), KCl (≥99%, ACS reagent), CsCl (≥99%, ACS reagent) and HNO_3_ (63%, ACS reagent) were purchased from Sigma-Aldrich (Austria) and used without further purification.

The POM salts Na_3_[PW_12_O_40_],^29,30^ K_4_[PVW_11_O_40_],^31^ Cs_5_[PV_2_W_10_O_40_],^31^ Cs_6_[PV_3_W_9_O_40_],^31^ Na_7_[PW_11_O_39_],^32^ H_4_[PVMo_11_O_40_],^33^ H_5_[PV_2_Mo_10_O_40_],^33^ H_6_[PV_3_Mo_9_O_40_],^33^ K_4_[SiW_12_O_40_],^34^ K_4_[SiMoW_11_O_40_],^35^ K_4_[SiMo_3_W_9_O_40_],^35^ and Na_2_K_4_[V_10_O_28_][Bibr ref36] were prepared according to reported procedures. All salts were characterized in solution by NMR spectroscopy and in the solid-state using infrared (IR, Figures S22–S24). Purity of all synthesized POM salts is ≥95%, as confirmed by NMR spectroscopy in aqueous solution, based on signal integration of POM species relative to total phosphorus- or vanadium-containing species detected.

### 4.1 NMR Spectroscopy

The speciation of PV_*x*_W_12–*x*_ and PV_*x*_Mo_12–*x*_ families was investigated using ^31^P and ^51^V NMR spectroscopy. The relative abundance of all species was calculated based on the integration of the ^31^P NMR peaks, considering only signals that can be assigned to POM species, ignoring free phosphate signals. For SiMo_*x*_W_12–*x*_ family ^183^W NMR has been applied. ^31^P, ^51^V, and ^183^W NMR spectra were recorded on a Avance Neo 500 MHz FT-NMR spectrometer (Bruker, Rheinstetten, Germany) at 25 °C. Chemical shifts were measured relative to 85% H_3_PO_4_, VOCl_3,_ and 1 M Na_2_WO_4_, respectively. The ^31^P NMR spectra were recorded at 202.53 MHz and the ^51^V NMR samples were recorded at 131.60 MHz (2000 scans, accumulation time 0.05 s, relaxation delay 0.01 s). The ^183^W NMR samples were prepared at a concentration of 20 mM in freshly prepared DMEM + 2% FCS + 50 mg/L gentamicin and measured in 10 mm NMR tubes.

The experimental time was about 60 h, with a standard pulse program at 20.836 MHz and a flip angle of 63° with a 1 s relaxation delay.

### 4.2 Antiviral Assays

The antiviral activity of the test compounds against HCoV-OC43 was determined according to a recently reported protocol.^37^ In short: A virus dilution at an MOI (multiplicity of infection) of 0.1 was simultaneously added with the test compounds to an almost confluent layer of RD cells. After 24 h the incubation was stopped and the viral load was quantified by ELISA using a N-protein primary antibody (N-protein HCoV-OC43, Sino Biological) and a HRP-labeled secondary antibody (Sino biological). Following the determination of N-positive cells, the plates were washed twice with phosphate buffered saline and a crystal violet counter screen was performed to determine cytotoxicity.

For time of-addition assays three treatment conditions were applied. For pretreatment, the test compounds were incubated with the cells for 1.5 h prior to infection. After 1.5 h the cells were washed with PBS twice and infected with HCoV-OC43 at a MOI of 0.1 for 1.5 h. After the infection period, cells were washed with PBS twice and incubated with fresh medium until 24 h post infection.

For cotreatment, the compounds were added simultaneously with the virus dilution of MOI 0.1 and incubated for 1.5 h. After 1.5 h the cells were washed with PBS twice and incubated with fresh medium until 24 h post infection.

For postinfection treatment cells were infected with HCoV-OC43 at an MOI of 0.1 for 1.5 h. After 1.5 h, cells were washed with PBS twice, the compounds were added and incubated until 24 h post infection. After stopping, viral load was determined as described above.

## Supplementary Material



## Data Availability

**Molecular Formula Strings:** the compounds reported in this study are inorganic polyoxometalate cluster anions for which SMILES representation is not standard. Molecular formulas and charges are provided in Table 1. The data sets containing NMR spectra for this study have been deposited in the database PHAIDRA (University of Vienna): https://phaidra.univie.ac.at/o:2337471

## Notes

PDF file includes Abbreviation list, Figures S1 to S24, Tables S1 and S2 (DOCX)
