# Supplementary material for: Keggin-Type Polyoxometalates as a Systematic Antiviral Scaffold: Activity, Cytotoxicity, and Solution Stability across Heteroion and Addenda Metal Variations
Source: J Med Chem. 2026 Aug 17;69(17):20772–81. doi: 10.1021/acs.jmedchem.6c01515 (PMC13576392; doi:10.1021/acs.jmedchem.6c01515)
Supplement: Supplementary file 1 [file jm6c01515_si_001.docx]

Supplementary Information

Keggin-Type Polyoxometalates as a Systematic Antiviral Scaffold: Activity, Cytotoxicity, and Solution Stability across Heteroion and Addenda Metal Variations

Judith Füllborn-Ott,^¶^ Aliona Dobrova,^†^ Nadiia I. Gumerova,^†^ Ingo Ott,*^¶^ Annette Rompel*^†^

^†^ Universität Wien, Fakultät für Chemie, Institut für Biophysikalische Chemie, 1090 Wien, Austria. www.bpc.univie.ac.at, Email: annette.rompel@univie.ac.at

^¶^ Institute of Medicinal and Pharmaceutical Chemistry, Technische Universität Braunschweig, Beethovenstr. 55, 38106 Braunschweig, Germany, Email: ingo.ott@tu-braunschweig.de

1. Abbreviation list

| ADMET | Absorption, Distribution, Metabolism, Excretion, and Toxicity | P_2_Mo_5_ | [P_2_Mo_5_O_23_]^6–^ |
| --- | --- | --- | --- |
| CC_50_ | 50% cytotoxic concentration | PVW_11_ | [PVW_11_O_40_]^4–^ |
| DMEM | Dulbecco's Modified Eagle's Medium | PV_2_W_10_ | [PV_2_W_10_O_40_]^5–^ |
| FCS | fetal calf serum | PV_3_W_9_ | [PV_3_W_9_O_40_]^6–^ |
| FDA | United States Food and Drug Administration | PVMo_11_ | [PVMo_11_O_40_]^4–^ |
| HCoV-OC43 | human coronavirus OC43 | PV_2_Mo_10_ | [PV_2_Mo_10_O_40_]^5–^ |
| IC_50_ | 50% inhibitory concentration | PV_3_Mo_9_ | [PV_3_Mo_9_O_40_]^6–^ |
| MOI | multiplicity of infection | SiW_12_ | [SiW_12_O_40_]^4–^ |
| NMR | nuclear magnetic resonance | SiMoW_11_ | [SiMoW_11_O_40_]^4–^ |
| PBS | phosphate buffer saline | SiMo_3_W_9_ | [SiMo_3_W_9_O_40_]^4–^ |
| POM | polyoxometalate | SARS-CoV-2 | severe acute respiratory syndrome coronavirus 2 |
| PW_12_ | [PW_12_O_40_]^3–^ | SD | standard deviation |
| PW_11_ | [PW_11_O_39_]^7–^ | V_10_ | [V_10_O_28_]^6–^ |
| PMo_12_ | [PMo_12_O_40_]^3–^ | V_2_–V_4_ | Small oligomeric vanadate species: divanadate [V_2_O_7_]^4–^, trivanadate [V_3_O_9_]^3–^, and tetravanadate [V_4_O_12_]^4–^ |
| PMo_11_ | [PMo_11_O_39_]^7–^ |  |  |

2. POM solution studies

# 2.1 pH behavior of POM solutions in water and cell culture medium

**Figure S1**. pH values of 1 mM solutions of the **PV_x_W_12–x_** (x = 0–3) and **PV_x_Mo_12–x_** (x = 0–3) series measured in H_2_O (grey), fresh DMEM + 2% FCS (Fetal Calf Serum) + 50 mg/L gentamicin (green), and after 24 h incubation at 37 °C in DMEM (orange).

# 2.2 NMR studies in PV_x_W_12–x_ series

**Figure S2**. ^31^P NMR spectra of 1 mM PW_12_ in H_2_O, fresh DMEM + 2% FCS (fetal calf serum) + 50 mg/L gentamicin, and after 24 h incubation at 37 °C in DMEM. Signals corresponding to individual POM species are indicated by filled circles.

**Figure S3**. ^31^P NMR spectra of 1 mM PVW_11_ in H_2_O, fresh DMEM + 2% FCS (fetal calf serum) + 50 mg/L gentamicin, and after 24 h incubation at 37 °C in DMEM. Signals corresponding to individual POM species are indicated by filled circles.

**Figure S4**. ^51^V NMR spectra of 1 mM PVW_11_ in H_2_O, fresh DMEM + 2% FCS (fetal calf serum) + 50 mg/L gentamicin, and after 24 h incubation at 37 °C in DMEM. Color code: W-centered octahedra (blue), V-substituted positions (orange), heteroion – P (gold). Signals corresponding to individual POM species are indicated by filled circles.

**Figure S5**. ^31^P NMR spectra of 1 mM PV_2_W_10_ in H_2_O, fresh DMEM + 2% FCS (fetal calf serum) + 50 mg/L gentamicin, and after 24 h incubation at 37 °C in DMEM. Signals corresponding to individual POM species are indicated by filled circles.

**Figure S6**. ^51^V NMR spectra of 1 mM PV_2_W_10_ in in H_2_O, fresh DMEM + 2% FCS (fetal calf serum) + 50 mg/L gentamicin, and after 24 h incubation at 37 °C in DMEM. Color code: W-centered octahedra (blue), V-substituted positions (orange), V in V_10_ (yellow), heteroion – P (gold). Signals corresponding to individual POM species are indicated by filled circles.

**Figure S7**. ^31^P NMR spectra of 1 mM PV_3_W_9_ in H_2_O, fresh DMEM + 2% FCS (fetal calf serum) + 50 mg/L gentamicin, and after 24 h incubation at 37 °C in DMEM. Color code: W-centered octahedra (blue), V-substituted positions (orange), heteroion – P (gold). Signals corresponding to individual POM species are indicated by filled circles; the color code is consistent throughout the figure.

**Figure S8**. ^51^V NMR spectra of 1 mM PV_3_W_9_ in H_2_O, fresh DMEM + 2% FCS (fetal calf serum) + 50 mg/L gentamicin, and after 24 h incubation at 37 °C in DMEM. Color code: W-centered octahedra (blue), V-substituted positions (orange), heteroion – P (gold). Signals corresponding to individual POM species are indicated by filled circles.

**Table S1**. Chemical shifts in ^31^P and ^51^V NMR spectra measured in triplicate for **PV_x_W_12–x_** series. pH values of all solutions under all three conditions are provided in Figure S1.

| POM | Figure | δ (^31^P), ppm | % of POM anions based on the ^31^P signal integration | Figure | δ (^51^V), ppm | % of POM anions based on the ^51^V signal integration |
| --- | --- | --- | --- | --- | --- | --- |
| 1 mM **PW_12_** in H_2_O | S2 | 0, –12.2, –12.6, –13.0, –13.9, –15.29 | 29.5 ± 7.8 % **PW_12_** | – | – | – |
| 1 mM **PW_12_** in fresh DMEM + 2% FCS + 50 mg/l gentamicin | S2 | 1.8, –11.8 | 100 % **PW_11_** | – | – | – |
| 1 mM **PW_12_** in DMEM + 2% FCS + 50 mg/l gentamicin after 24 h at 37 °C | S2 | 2.0, –11.8 | 100 % **PW_11_** | – | – | – |
| 1 mM **PVW_11_** in H_2_O | S3 | –14.9 | 100 % **PVW_11_** | S4 | –555.7 | 100 % **PVW_11_** |
| 1 mM **PVW_11_** in fresh DMEM + 2% FCS + 50 mg/l gentamicin | S3 | only 2.0 from free phosphate |  | S4 | –559.1 (broad) | 100 % **PVW_11_** |
| 1 mM **PVW_11_** in DMEM + 2% FCS + 50 mg/l gentamicin after 24 h at 37 °C | S3 | 2.0, –10.9, –11.1, –11.5, –11.8 | 100 % **PVW_11_** | S4 | –557.2 (broad) | 100 % **PVW_11_** |
| 1 mM **PV_2_W_10_** in H_2_O | S5 | 0.3, –10.9, –12.5, –13.6, –14.33 | 25.1±0.9 % α-**PV_2_W_10_**  31.7±6.5 % β-**PV_2_W_10_**  17.7±1.9 % γ-**PV_2_W_10_**  25.5±5.5 % PW11 | S6 | –422.2, –498.4, –519.6, –526.5, –543.2, –546.9, –553.4, –557.6, –558.2, –579.7 | – |
| 1 mM **PV_2_W_10_** in fresh DMEM + 2% FCS + 50 mg/l gentamicin | S5 | only 2.0 from free phosphate | – | S6 | -420.5, –494.8, –509.2, –570.0, –575.0 | 31.7±5.5 % **V_10_** |
| 1 mM **PV_2_W_10_** in DMEM + 2% FCS + 50 mg/l gentamicin after 24 h at 37 °C | S5 | 2.13, –11.2 | 100 % **PW_11_** | S6 | –509.1, –551.8, –558.8, –570.0, –575.0 | 23.7±5.1 % **VW_5_** |
| 1 mM **PV_3_W_9_** in H_2_O | S7 | –12.3, –13.5, –14.3 | 91.1±0.9 % **PV_3_W_9_** | S8 | –509.0, –531.0, –546.8, –558.3 | 81.9±3.6 % **PV_3_W_9_** |
| 1 mM **PV_3_W_9_** in fresh DMEM + 2% FCS + 50 mg/l gentamicin | S7 | 2.3, –13.5 | 100 % **PV_3_W_9_** | S8 | –531.2, –557.7, –569.3, –575.0 | 62.1±5.0 % **PV_3_W_9_** |
| 1 mM **PV_3_W_9_** in DMEM + 2% FCS + 50 mg/l gentamicin after 24 h at 37 °C | S7 | 2.2, –11.2, –12.5, –13.5 | 92.1±6.9 % **PV_3_W_9_** | S8 | –509.2, –531.2, –558.4, –569.7, –575.0, –583.2 | 57.8±1.8 % **PV_3_W_9_** |

# 2.3 NMR studies in PV_x_Mo_12–x_ series

**Figure S9**. ^31^P NMR spectra of 1 mM PMo_12_ in H_2_O, fresh DMEM + 2% FCS (fetal calf serum) + 50 mg/L gentamicin, and after 24 h incubation at 37 °C in DMEM. Color code: Mo-centered octahedra (teal), heteroion – P (gold). Signals corresponding to individual POM species are indicated by filled circles.

**Figure S10**. ^31^P NMR spectra of 1 mM PVMo_11_ in H_2_O, fresh DMEM + 2% FCS (fetal calf serum) + 50 mg/L gentamicin, and after 24 h incubation at 37 °C in DMEM. Color code: Mo-centered octahedra (teal), V-substituted positions (orange), heteroion – P (gold). Signals corresponding to individual POM species are indicated by filled circles; the color code is consistent throughout the figure.

**Figure S11**. ^51^V NMR spectra of 1 mM PVMo_11_ in H_2_O, fresh DMEM + 2% FCS (Fetal Calf Serum) + 50 mg/L gentamicin, and after 24 h incubation at 37 °C in DMEM. Color code: Mo-centered octahedra (teal), V-substituted positions (orange), heteroion – P (gold). Signals corresponding to individual POM species are indicated by filled circles; the color code is consistent throughout the figure.

**Figure S12**. ^31^P NMR spectra of 1 mM PV_2_Mo_10_ in H_2_O, fresh DMEM + 2% FCS (fetal calf serum) + 50 mg/L gentamicin, and after 24 h incubation at 37 °C in DMEM. Color code: Mo-centered octahedra (teal), V-substituted positions (orange), heteroion – P (gold). Signals corresponding to individual POM species are indicated by filled circles; the color code is consistent throughout the figure.

**Figure S13**. ^51^V NMR spectra of 1 mM PV_2_Mo_10_ in H_2_O, fresh DMEM + 2% FCS (fetal calf serum) + 50 mg/L gentamicin, and after 24 h incubation at 37 °C in DMEM. Color code: Mo-centered octahedra (teal), V-substituted positions (orange), heteroion – P (gold). Signals corresponding to individual POM species are indicated by filled circles; the color code is consistent throughout the figure.

**Figure S14**. ^31^P NMR spectra of PV_3_Mo_9_ in H_2_O, fresh DMEM + 2% FCS (fetal calf serum) + 50 mg/L gentamicin, and after 24 h incubation at 37 °C in DMEM. Color code: Mo-centered octahedra (teal), V-substituted positions (orange), heteroion – P (gold). Signals corresponding to individual POM species are indicated by filled circles; the color code is consistent throughout the figure.

**Figure S15**. ^51^V NMR spectra of 1 mM PV_3_Mo_9_ in H_2_O, fresh DMEM + 2% FCS (fetal calf serum) + 50 mg/L gentamicin, and after 24 h incubation at 37 °C in DMEM. Color code: Mo-centered octahedra (teal), V-substituted positions (orange), heteroion – P (gold). Signals corresponding to individual POM species are indicated by filled circles; the color code is consistent throughout the figure.

**Table S2**. Chemical shifts in ^31^P and ^51^V NMR spectra measured in triplicate for **PV_x_Mo_12–x_** series. pH values of all solutions under all three conditions are provided in Figure S1.

| POM | figure | δ (^31^P), ppm | % of POM anions based on the ^31^P signal integration | figure | δ (^51^V), ppm | % of POM anions based on the ^51^V signal integration |
| --- | --- | --- | --- | --- | --- | --- |
| 1 mM **PMo_12_** in H_2_O | S9 | 2.0, 0.9, 0.7,  –1.4 | 18.6 ± 3.6 % **PMo_11_** | – | – | – |
| 1 mM **PMo_12_** in fresh DMEM + 2% FCS + 50 mg/l gentamicin | S9 | 3.4, 2.7 | 100 % **P_2_Mo_5_** | – | – | – |
| 1 mM **PMo_12_** in DMEM + 2% FCS + 50 mg/l gentamicin after 24 h at 37 °C | S9 | 1.0, 0.1 | 100 % **P_2_Mo_5_** | – | – | – |
| 1 mM **PVMo_11_** in H_2_O | S10 | 0.1, –1.6, –4.2 | 97.1 ± 1.6 % **PVMo_11_** | S11 | –520.1,  –531.5,  –532.8,  –533.2 | 94.6 ± 0.4 % **PVMo_11_** |
| 1 mM **PVMo_11_** in fresh DMEM + 2% FCS + 50 mg/l gentamicin | S10 | 1.0, 0.2, –4.3 | The peak intensities are too low for the integration. | S11 | –496.8,  –513.0,  –532.2,  –562.2 | 74.8 ± 1.8 % **PVMo_11_** |
| 1 mM **PVMo_11_** in DMEM + 2% FCS + 50 mg/l gentamicin after 24 h at 37 °C | S10 | 1.0, 0.2, –4.3 | The peak intensities are too low for the integration. | S11 | –496.6,  –512.2, –529.5, –532.8 | 80.6 ± 1.0 % **PVMo_11_** |
| 1 mM **PV_2_Mo_10_** in H_2_O | S12 | –3.3, –3.4,  –3.5, –3.7,  –4.2, –4.3 | 29.9 ± 4.5 % **PV_2_Mo_10_**  70.0 ± 4.6 % **PVMo_11_** | S13 | –519.6, –531.5, –532.7, –533.1, –534.5, –536.7, –538.9, –540.7, –541.9, –543.9, –547.1 | 56.9 ± 0.7 % (**PVMo_11_** + **PV_2_Mo_10_**) |
| 1 mM **PV_2_Mo_10_** in fresh DMEM + 2% FCS + 50 mg/l gentamicin | S12 | 0.5, –4.3 | 100 % **PVMo_11_** | S13 | –495.3, –512.4, –519.6, –531.9, –560.3, –570.7, –575.1 | 37.6 ± 0.7 % (**PVMo_11_** + **PV_2_Mo_10_**) |
| 1 mM **PV_2_Mo_10_** in DMEM + 2% FCS + 50 mg/l gentamicin after 24 h at 37 °C | S12 | 0.4, –4.3 | 100 % **PVMo_11_** | S13 | –496.3, –511.2, –512.7, –531.8, –560.9, –571.0, –575.1 | 35.3 ± 2.6 % (**PVMo_11_** + **PV_2_Mo_10_**) |
| 1 mM **PV_3_Mo_9_** in H_2_O | S14 | 0.1, –2.7,  –2.8, –3.1,  –3.3, –3.4, –3.5, –3.6, –3.7, –4.1, –4.2, –4.2 | 42.1 ± 2.3 % **PVMo_11_**, 57.9 ± 2.3 % (**PV_2_Mo_10_** + **PV_3_Mo_9_**) | S15 | –519.3, –531.2, –533.0, –534.3, –535.0, –533.3, –538.5, –540.1, –540.5, –541.7, –543.6, –546.9, –548.5 | 41.3 ± 1.9 % (**PVMo_11_** + **PV_2_Mo_10_** + **PV_3_Mo_9_**) |
| 1 mM **PV_3_Mo_9_** in fresh DMEM + 2% FCS + 50 mg/l gentamicin | S14 | 0.5, –4.3 | 100 % **PVMo_11_** | S15 | –512.3, –519.6, –530.6, –560.3, –570.8, –580.7 | 30.7 ± 1.5 % (**PVMo_11_** + **PV_2_Mo_10_** + **PV_3_Mo_9_**) |
| 1 mM **PV_3_Mo_9_** in fresh DMEM + 2% FCS + 50 mg/l gentamicin after 24 h at 37 °C | S14 | 0.3 (free phosphate) | – | S15 | –422.8, –496.2, –502.7, –511.3, –512.7, –514.2, –523.2, –531.5, –560.7, –571.2, –575.1 | 19.9 ± 2.2 % (**PVMo_11_** + **PV_2_Mo_10_** + **PV_3_Mo_9_**) |

3. Antiviral activity and cytotoxicity determination

**Figure S16.** Dose-response curves for the PV_x_W_12–x_ (x = 0–3) series. Antiviral activity against HCoV-OC43 (green, left y-axis) and cytotoxicity against host cells (blue, right y-axis) are shown as a function of compound concentration (logarithmic scale). Data points represent mean ± SD (n = 3–4).

**Figure S17.** Dose-response curves for the PV_x_Mo_12–x_ (x = 0–3) series. Antiviral activity against HCoV-OC43 (green, left y-axis) and cytotoxicity against host cells (blue, right y-axis) are shown as a function of compound concentration (logarithmic scale). Data points represent mean ± SD (n = 3–4).

**Figure S18.** Dose-response curves for the SiMo_x_W_12–x_ series. Antiviral activity against HCoV-OC43 (green, left y-axis) and cytotoxicity against host cells (blue, right y-axis) are shown as a function of compound concentration (logarithmic scale). Data points represent mean ± SD (n = 3–4).

**Figure S19.** Dose-response curves for lacunary POM derivatives and the decavanadate control. Antiviral activity against HCoV-OC43 (green, left y-axis) and cytotoxicity against host cells (blue, right y-axis) are shown as a function of compound concentration (logarithmic scale). Data points represent mean ± SD (n = 3–4).

**Figure S20.** Dose-response curves for simple metal salt controls. Antiviral activity against HCoV-OC43 (green, left y-axis) and cytotoxicity against host cells (blue, right y-axis) are shown as a function of compound concentration (logarithmic scale). Data points represent mean ± SD (n = 3–4).

**Figure S21.** Dose-response curves for reference compounds K_7_[PTi_2_W_10_O_40_] (left) and K_11_H[(VO)_3_(SbW_9_O_33_)_2_]. Antiviral activity against HCoV-OC43 (green, left y-axis) and cytotoxicity against host cells (blue, right y-axis) are shown as a function of compound concentration (logarithmic scale). Data points represent mean ± SD (n = 3–4).

4. IR Spectroscopy

**Figure S22.** IR spectra of PV_x_W_12–x_ family (x = 0–3) and decomposition products PW_11_ and V_10_ investigated in this study.

**Figure S23.** IR spectra of PV_x_Mo_12–x_ family (x = 0–3) investigated in this study.

**Figure S24.** IR spectra of SiMo_x_W_12–x_ family (x = 0, 1, 3) investigated in this study.
